# Factors Associated with COVID-19 Vaccine Intentions in Eastern Zimbabwe: A Cross-Sectional Study

**DOI:** 10.3390/vaccines9101109

**Published:** 2021-09-29

**Authors:** Lauren McAbee, Oscar Tapera, Mufaro Kanyangarara

**Affiliations:** 1 Department of Epidemiology and Biostatistics, Arnold School of Public Health, University of South Carolina, Columbia, SC 29208, USA; lgmcabee@email.sc.edu; 2 SADTAP Health Research Institute, Harare 00263, Zimbabwe; oscar.tapera@gmail.com

**Keywords:** COVID-19 vaccine, vaccine intentions, COVID-19 pandemic, Zimbabwe, Sub-Saharan Africa

## Abstract

Vaccines are one of the most effective public health strategies to protect against infectious diseases, yet vaccine hesitancy has emerged as a global health threat. Understanding COVID-19 knowledge and attitudes and their association with vaccine intentions can help the targeting of strategies to increase vaccination uptake and achieve herd immunity. The goal of this study was to assess COVID-19 knowledge, attitudes, and behaviors, and identify factors associated with COVID-19 vaccine intentions among heads of households in Manicaland Province, Zimbabwe. A cross-sectional survey was conducted in May 2021 among 551 randomly selected households. Data were collected on socio-demographic characteristics, and knowledge, attitudes, and behaviors regarding COVID-19 and the vaccines. More than half (55.7%) of the respondents reported intending to vaccinate themselves or their households. Multivariate logistic regression indicated that the likelihood of vaccine intentions was most strongly associated with confidence in vaccine safety. Additionally, the odds of intending to get vaccinated were significantly higher among heads of households who were male, had a higher level of education, and identified vaccination and face mask usage as prevention measures. Among perceived motivators to vaccinate, recommendations from the World Health Organization and availability of the vaccine free of charge increased the likelihood of vaccine intentions, while country of vaccine manufacturer posed a barrier to vaccine intentions. As the vaccine rollout in Zimbabwe continues, efforts to increase COVID-19 vaccination coverage and achieve herd immunity should target females and less educated populations and be tailored to address concerns about vaccine safety and country of manufacturer.

## 1. Introduction

Severe acute respiratory syndrome coronavirus-2 (SARS-CoV-2), which causes coronavirus disease 2019 (COVID-19), has resulted in over 218 million cases and 4.5 million deaths globally [1]. To reduce the spread of COVID-19, the World Health Organization (WHO) recommends wearing face masks, frequent handwashing, social distancing, covering coughs and sneezes, disinfecting high-touch areas daily, and quarantining or isolating when appropriate [2]. The approval of several COVID-19 vaccines for emergency use has added to the arsenal against COVID-19 and represents a huge step toward the return to normalcy. Despite the increasing availability of COVID-19 vaccines globally, an ever-growing concern in global health is the equitable distribution of and access to vaccines, especially in low- and middle-income countries. To promote COVID-19 vaccine equity, the COVAX facility has targeted to distribute more than 2 billion doses globally by the end of 2021, with the goal of reaching 20% population coverage [3]. To date, 234 million doses have been distributed through the COVAX facility [4]. Nevertheless, many low- and middle-income countries still face challenges in the equitable distribution of COVID-19 vaccines because of lack of vaccine manufacturing capacity, fragile healthcare systems, logistical challenges with transporting and storing vaccines, shortages in health care workers, and poor access to essential health services [5,6,7]. Efforts to reduce the burden of COVID-19 through vaccination are further compounded by the emerging threat of vaccine hesitancy [8]. 

Vaccine hesitancy is defined as the “delay in acceptance or refusal of vaccination despite availability of vaccination services” [9]. Vaccine hesitancy can drive outbreaks of vaccine-preventable diseases, lead to slower vaccination rates, and hinder the attainment and sustainability of herd immunity. The 2003–2004 polio vaccine boycott in Nigeria, resistance towards the oral cholera vaccination in Mozambique, and continued objections to routine childhood vaccinations by members of the Apostolic sect in Zimbabwe all illustrate the negative public health impact of vaccine hesitancy [10,11,12].

Determinants of vaccine hesitancy can be categorized as confidence in the effective-ness and safety of vaccines and trust in the health care system and policy makers, complacency about the vaccine, and convenience which encompasses physical availability, affordability, willingness to pay, and accessibility [9]. Several studies in Africa have identified key drivers and barriers of the uptake of routine child and adolescent vaccination and these include concerns about efficacy and safety, mistrust of the healthcare system, religious beliefs, low socioeconomic status, lack of knowledge of the vaccine and its effectiveness, rural residence, and poor access to immunization services [12,13,14,15].

Recent studies focused on the uptake of COVID-19 vaccines have identified similar factors with the major barriers being concerns about safety and side effects, and a general lack of trust in governments and the pharmaceutical companies that developed them [16,17,18,19,20,21]. A study by the Africa Centers for Disease Control and Prevention found that willingness to accept COVID-19 vaccines across 15 African countries ranged from 59% to 94% [21]. Individuals who were younger, unemployed, living in urban cities, and who disagreed vaccines were safe were more likely to be skeptical about COVID-19 vaccines. This study found that women were more skeptical about COVID-19 vaccines, which is consistent with other studies that have identified gender differences in vaccine confidence and acceptability [21,22]. Socio-cultural and religious beliefs also play a significant role in COVID-19 vaccine acceptance in Africa, as the majority of the population is religious [23,24]. One West African study found that most participants believed that prayer was more effective against COVID-19 than the vaccine [24]. Vaccine acceptance has also been hindered by the spread of misinformation not only by unqualified individuals but by religious, political, and community leaders [25]. With most concerns about vaccines being centered around the safety and efficacy of the vaccines, it is vital that accurate information is spread so that vaccines can be distributed and administered appropriately globally.

Assessing vaccine intentions and identifying barriers and motivators of COVID-19 vaccine intentions can guide the targeting of evidence-based interventions to populations and areas most at risk. The objective of this study was to understand the knowledge, behaviors, and attitudes about COVID-19 and determine factors associated with COVID-19 vaccine intentions in Manicaland, Zimbabwe. In Zimbabwe, there have been over 125,000 cases of COVID-19 and at least 4450 deaths due to COVID-19 to date [26]. Zimbabwe was one of the first African countries to receive COVID-19 vaccines, and a vaccine rollout plan was implemented in February 2021 [25]. Zimbabwe has administered 4.3 million doses of vaccines and about 17.5% of the population has received at least one dose of a COVID-19 vaccine, as of August 2021 [26]. However, there is a paucity of research in Zimbabwe to understand COVID-19 vaccine intentions and associated factors [20,25,27]. A national online survey conducted in February 2021, when vaccines were not yet available in the country, reported vaccine intentions among half of Zimbabweans [27]. However, as the online survey consisted of a sample of primarily urban residents of Harare with internet access, the study findings are not generalizable to rural areas lacking access to the internet. Given the likely subnational variation in vaccine intentions, this study fills a critical and timely gap in understanding subnational vaccine intentions in Zimbabwe. This study builds on previous research by considering vaccine intentions when vaccines were available in a primarily rural province, that has historically had lower vaccination coverages rates for childhood illnesses. Consequently, it is expected that residents of this province will more likely exhibit increased hesitancy towards a new vaccine.

## 2. Materials and Methods

### 2.1. Study Design and Setting

This secondary data analysis was based on a population-based survey conducted as part of a larger study to investigate the impact of the COVID-19 pandemic on malaria transmission and control in Zimbabwe. Zimbabwe has a total population of about 15 mil-lion, and 38% of the population lives in urban areas [28]. In May 2021, when the survey was conducted, there were more than 37,000 confirmed COVID-19 cases and 1500 deaths nationally [1]. Manicaland Province had the third highest number of cumulative cases, after Harare and Bulawayo provinces [1]. There was a mask mandate in place, requiring face masks in public, but the country was not under lockdown, as most businesses and schools were allowed to open. Only 2% of the population in the country had received at least one dose of a COVID-19 vaccine [26].

Three districts were randomly selected to represent high, moderate and low malaria transmission. Mutare, Makoni and Buhera districts represent 45% of the population of Manicaland Province. Using enumeration areas from the 2012 census as a sampling frame, 551 households were randomly selected from the three selected districts to complete the single-visit household survey. The sample size required for the larger study was based on detecting a 10% difference in health-seeking behaviors between districts assuming 80% power and an alpha of 0.05.

Interviewers approached selected households and explained the study purpose and procedures. The inclusion criteria for study participants were being 18 years of age or older, head of household or caregiver, and willing and able to provide consent. After obtaining written informed consent, interviewers read aloud each question to the participants and recorded responses on a mobile device programmed with SurveyToGo software (Dooblo Ltd., Kefar Sava, Israel). The questionnaire took about 45 min to complete for each participant. Data were collected on characteristics of the household and the head, COVID-19 knowledge, attitudes, and behaviors, risk perceptions, utilization of health services, vaccination status, vaccination intentions, and confidence in safety of the COVID-19 vaccines and motivators and barriers influencing the decision to get vaccinated.

### 2.2. Statistical Analysis

Socio-demographic characteristics of the respondents and their households were described using descriptive statistics. The outcome of interest was to get vaccinated against COVID-19 at the household level. Univariate and multivariate logistic regression analyses were conducted to identify factors associated with vaccination intentions. Fac-tors assessed included household head age, gender, education level, level of confidence in vaccine safety, knowledge of COVID-19 symptoms and prevention measures, reported practice of COVID-19 prevention measures, and attitudes toward COVID-19. Variables with a *p*-value of <0.2 in the univariate analysis were included in the initial multivariate analysis. Backwards elimination was then used to retain statistically significant variables (*p* < 0.05) in the multivariate model. Measures of association were presented as unadjusted and adjusted odds ratios and 95% confidence intervals. STATA/BE 17.0 software (College Station, TX, US) was used to conduct all analyses.

### 2.3. Ethical Considerations

Ethical approval was obtained from the Medical Research Council of Zimbabwe (MRCZ/2633). Local leaders and community members were informed about the study and permission was sought to conduct the study in their communities. Written informed con-sent was obtained from study participants prior to data collection. Wearing face masks, social distancing, and frequent handwashing or sanitization were practiced during data collection to curb the spread of COVID-19.

## 3. Results

### 3.1. Sociodemographic Characteristics

Of the 551 survey responses, 103 (18.7%) households had at least one member who had been vaccinated against COVID-19, 307 (55.7%) households intended to get vaccinated, and 141 (25.6%) did not intend to get vaccinated (Table 1). Most respondents were female (72.4%), less than 55 years old (75.3%), married or co-habiting (67.5%), employed (58.4%), had attended secondary school or higher (68.8%), and had at least one child under 5 years of age living in the household (56.1%). Very few (1.5%) households had a member who had been diagnosed with COVID-19. There were significant differences in sociodemographic characteristics by vaccination status. Heads of households with at least one vaccinated member were more likely to be employed, have attended secondary school or higher, have health insurance, reside in Mutare rural district, and be near a health facility. Among heads of households with no vaccinated members, vaccination intentions were more likely among those who were younger, less educated, female, and in Buhera district.

### 3.2. COVID-19 Knowledge, Attitudes, and Behaviors

**Figure 1:**
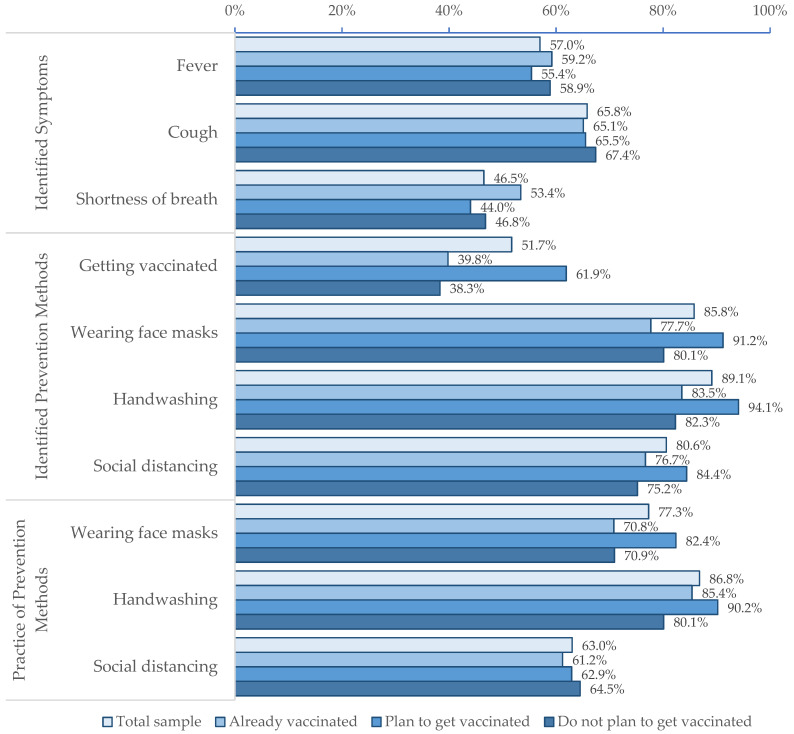
Knowledge, attitudes, and behaviors regarding COVID-19 and the COVID-19 vaccines, categorized by vaccination status.

Although more than half of respondents identified fever (57.0%) and cough (65.8%) as common COVID-19 symptoms, fewer than half (46.5%) were able to identify shortness of breath (Figure 1). Most respondents identified handwashing (89.1%), social distancing (80.6%), and wearing face masks (85.8%) as ways to prevent the risk of COVID-19 infection, but fewer reported practicing these measures (86.8%, 63.0%, and 77.3%, respectively). The knowledge of prevention measures and reported practice of these measures were consistently higher among respondents with vaccine intentions compared to those without. Although concerns about community spread of COVID-19 and acquiring infection were similar across households regardless of vaccine intentions, there were substantial differences in confidence in vaccine safety. About two-thirds (68.7%) of respondents with vaccine intentions were confident that COVID-19 vaccines were completely safe compared to 8.5% of those with no intention of getting vaccinated.

### 3.3. Factors Associated with Vaccine Intentions

In the univariate analysis, the odds of intending to get vaccinated were higher among respondents who had attended secondary school or higher (OR 2.04, 95% CI 1.35–3.09), reported handwashing (OR 2.29, 95% CI 1.31–4.00) and wearing face masks (OR 1.92, 95% CI 1.20–3.07), and were confident that COVID-19 vaccines were completely safe (OR 23.63, 95% CI 12.47–44.76) (Table 2). Knowledge of vaccination (OR 2.62, 95% CI 1.74–3.94), wearing face masks (OR 2.57, 95% CI 1.45–4.55), handwashing (OR 3.46, 95% CI 1.82–6.58), and social distancing (OR 1.78, 95% CI 1.09–2.91) as prevention measures increased the odds of intending to get vaccinated. Significant factors influencing the decision to get vaccinated included whether the World Health Organization (OR 2.88, 95% CI 1.56–5.31) or the Ministry of Health (OR 2.97, 95% CI 1.96–4.49) recommended vaccination, whether the vaccine was free of charge (OR 3.63, 95% CI 2.26–5.85), and ease of getting the vaccine (OR 2.94, 95% CI 1.82–4.74). Household size, district of residence, employment status of head of household, marital status of head of household, knowledge of COVID-19 symptoms, previous diagnosis of a household member with COVID-19, and concerns about community spread or getting infected with COVID-19 were not associated with vaccine intentions.

The multivariate logistic regression results showed that the odds of intending to get vaccinated were most strongly associated with confidence in vaccine safety (aOR 28.14, 95% CI 13.89–57.01). Being male (aOR 2.63, 95% CI 1.41–4.76), having attended secondary school or higher (aOR 1.73, 95% CI 0.93–3.22), and knowledge of vaccination (aOR 1.75, 95% CI 1.02–2.98) and handwashing (aOR 3.50, 95% CI 1.42–8.67) as prevention measures increased the likelihood of intending to get vaccinated. Other factors that were associated with increased odds of intending to get vaccinated included recommendations from the World Health Organization (aOR 3.12, 95% CI 1.41–6.92) and the availability of vaccines free of charge (aOR 3.83, 95% CI 2.05–7.15).

## 4. Discussion

This study assessed COVID-19 knowledge, attitudes, and behaviors and identified factors associated with vaccine intentions among a random sample of 551 household heads in Manicaland Province, Zimbabwe. About half of the respondents were able to identify fever, cough, and shortness of breath as symptoms associated with COVID-19. More than 80% of respondents were able to identify wearing face masks, handwashing, and social distancing as preventive measures. Notably, fewer (51.7%) respondents identified vaccination as a preventive measure despite the roll-out of COVID-19 vaccines in Zimbabwe since February 2021. Among heads of households who had not received the COVID-19 vaccine, 68.5% reported intentions to vaccinate themselves and their households against COVID-19. Vaccine intentions were associated with being male, more educated, knowledgeable about COVID-19 prevention measures, and confident in the safety of COVID-19 vaccines. As the COVID-19 vaccine roll-out in Zimbabwe continues, the findings from this study highlight several considerations in mounting evidence-based, tailored strategies to address vaccine hesitancy and improve vaccination coverage.

First, in this study, confidence in vaccine safety demonstrated the strongest association with vaccine intentions. Heads of households who were confident that COVID-19 vaccines were completely safe were 28 times more likely to intend to get vaccinated compared to those who lacked confidence in the safety of COVID-19 vaccines. These results are consistent with findings in other African settings that indicate that COVID-19 vaccine safety is a major concern and determinant of vaccine intentions and uptake [16,19,20,27]. Prior research on childhood vaccines has similarly demonstrated an association between perceived vaccine safety and vaccine intentions or uptake [12,15]. When Zimbabwe received its first shipment of doses from China, there was speculation in the local media about the safety of Sinopharm and its efficacy against the beta variant [29,30,31]. Furthermore, reported refusals to receive Chinese-made vaccines by health care workers and politicians received extensive attention in the media and may have influenced public trust in the safety of the vaccines [31]. Media attention highlighting the opposition to COVID-19 vaccines by these influential leaders may have consequently impacted vaccine intentions and undermined the trust and confidence placed in health care systems, governments and pharmaceutical companies manufacturing the vaccines. Health education targeting the safety and efficacy of the vaccine will most likely impact vaccine intentions and eventual uptake, especially if messaging comes from trusted sources.

In addition to safety, the present study found that cost and recommendations by the Ministry of Health and World Health Organization played a significant role in motivating vaccine intentions. Cost is unlikely to be a structural barrier associated with low vaccination rates in this context as the government of Zimbabwe has committed to making COVID-19 vaccines available free of charge to target population groups. However, reinforcing this information to the public may help increase vaccine acceptance. Since February 2021, the country has received 4.3 million doses of COVID-19 vaccines from China, India, and Russia [26]. Acceptance of COVID-19 vaccines has been slow and marred with distrust of Chinese-made vaccines, misinformation, and reluctance towards the vaccines from health care workers and government officials [25,29,30,31]. Distrust in the vaccines made by China and Russia has been reported in other countries [18,32]. However, given the high levels of trust in the Ministry of Health and World Health Organization as sources of information in this population, it is likely that the approval of the Chinese vaccines for emergency use by the World Health Organization will positively influence vaccine uptake in Zimbabwe [33].

This present study also demonstrated COVID-19 knowledge was significantly associated with vaccine intentions. Respondents with vaccine intentions were more likely to identify COVID-19 prevention measures compared to those without. Specifically, respondents who knew vaccination and handwashing were prevention measures were twice as likely to intend to get vaccinated. The reported practice of these prevention measures was more prevalent among those who intended to be vaccinated. Increased awareness and knowledge about COVID-19 is likely to increase motivation to get vaccinated and compliance with handwashing, face mask-wearing, and social distancing. Given that vaccine intentions in the present study were suboptimal among the older, female, and less educated, health education and sensitization should target these populations. Misinformation about vaccines can quickly spread and these populations may be more vulnerable to believing and spreading misinformation about COVID-19 vaccines.

There are a few limitations worth noting. First, causal inferences cannot be made as the study design was cross-sectional. Second, given the rapidly changing COVID-19 landscape, it is likely that the observed patterns of attitudes towards the COVID-19 vaccines and vaccine intentions will change over time. Third, the study was based on self-reported measures that are prone to social desirability bias and may not be indicative of actual behavior in the future. Fourth, while this study provides some insight on factors influencing vaccine intentions, there are likely additional factors that were not considered. While not addressed in the present study, religion may be driving the observed patterns in vaccine intentions and motivations. More than a third of the population belongs to the Apostolic sect which views vaccines as dangerous and causing disease and death [25]. Members of the sect have reported fearing being sanctioned and judged by other sect members for getting vaccinated [12]. Vaccination rates among sect members have consistently been lower than the rest of the population [34]. Given the long history of low vaccination rates for childhood and other adult vaccinations, the population is likely to have hesitancy towards receiving a relatively new vaccine. Lastly, given likely subnational variations in vaccine intentions, the findings of this study, while representative of Manicaland Province, may not be generalizable to other provinces in Zimbabwe or countries.

Despite the limitations, this study used a large sample of heads of households that provides a snapshot of vaccine intentions in Manicaland province. As vaccine availability becomes more widespread in Zimbabwe, the observed vaccine intentions could be realized soon if vaccines remain available and accessible. However, the findings of the present study underscore the need for tailored vaccination campaigns and education to effectively reach those with no intentions to vaccinate. Targeting women and less educated populations, coupled with sensitization about vaccine safety and vaccine manufacturer from trusted sources of information, including the Ministry of Health and World Health Organization, will help increase vaccine uptake. Additional studies exploring trends in vaccine uptake and intentions will be important to support efforts to achieve high levels of vaccination coverage and reduce the burden of COVID-19 in Zimbabwe.

## Figures and Tables

**Table 1:** Characteristics of households in Manicaland Province, Zimbabwe categorized by vaccination status.

Characteristic	Total	Vaccinated	Intend to Vaccinate	Do Not Intend to Vaccinate	*p*-Value
N	551	103	307	141	
Household head age					0.4
18–29 years	137 (24.9%)	24 (23.3%)	81 (26.4%)	32 (22.7%)	
30–39 years	136 (24.7%)	23 (22.3%)	84 (27.4%)	29 (20.6%)	
40–54 years	142 (25.8%)	24 (23.3%)	84 (27.4%)	34 (24.1%)	
≥55 years	136 (24.7%)	32 (31.1%)	58 (18.9%)	46 (32.6%)	
Household head is male	152 (27.6%)	28 (27.2%)	98 (31.9%)	115 (81.6%)	0.9
Household head is married/co-habiting	372 (67.5%)	62 (60.2%)	213 (69.4%)	97 (68.8%)	0.08
Household head attended secondary school or higher	379 (68.8%)	81 (78.6%)	220 (71.7%)	78 (55.3%)	0.02
Household head is employed	322 (58.4%)	74 (71.8%)	168 (54.7%)	80 (56.7%)	0.002
Household has more than 4 members	298 (54.1%)	50 (48.5%)	170 (55.4%)	78 (55.3%)	0.2
District of residence					0.008
Buhera	211 (38.3%)	27 (26.2%)	123 (40.1%)	61 (43.3%)	
Makoni	119 (21.6%)	22 (21.4%)	64 (20.9%)	33 (23.4%)	
Mutare rural	221 (40.1%)	54 (52.4%)	120 (39.1%)	47 (33.3%)	
Has health insurance	26 (4.7%)	13 (12.6%)	6 (2.0%)	7 (5.0%)	<0.001
Transportation time to get to health facility					0.002
0–45 min	363 (55.9%)	77 (74.7%)	189 (61.6%)	97 (68.8%)	
46–90 min	115 (20.9%)	18 (17.5%)	73 (23.8%)	24 (17.0%)	
≥90 min	73 (13.3%)	8 (7.8%)	45 (14.7%)	20 (14.2%)	
Drivers of decision to get vaccinated					
Recommendation from Ministry of Health	356 (64.6%)	68 (66.0%)	222 (72.3%)	66 (46.8%)	0.7
Recommendation from doctor	285 (51.7%)	66 (64.1%)	152 (49.5%)	67 (47.5%)	0.005
Vaccine available free of charge	210 (38.1%)	41 (39.8%)	142 (46.3%)	27 (19.2%)	0.7
Ease of getting the vaccine	195 (35.4%)	42 (40.8%)	126 (41.0%)	27 (19.2%)	0.2
Recommendation from World Health Organization	117 (21.2%)	29 (28.2%)	74 (24.1%)	14 (9.9%)	0.06
Country where vaccine is produced	102 (18.5%)	24 (23.3%)	39 (12.7%)	39 (27.7%)	0.2

**Table 2:** Factors influencing vaccine intentions among heads of households in Manicaland Province.

	Univariate			Multivariate		
	OR	95% CI	*p*-Value	aOR	95% CI	*p*-Value
Household head age						
18–29 years	Ref			Ref		
30–39 years	1.14	0.64–2.06	0.7	1.49	0.69–3.19	0.3
40–54 years	0.98	0.55–1.73	0.9	1.67	0.79–3.53	0.2
≥55 years	0.50	0.28–0.88	0.02	1.35	0.58–3.13	0.5
Household head is male	2.08	1.27–3.33	0.003	2.63	1.41–4.76	0.002
Household head attended secondary school or higher	2.04	1.35–3.09	0.001	1.73	0.93–3.22	0.08
Identification of COVID-19 prevention measures						
Getting vaccinated	2.62	1.74–3.94	<0.001	1.75	1.02–2.98	0.04
Handwashing	3.46	1.82–6.58	<0.001	3.51	1.42–8.67	0.007
Wearing face masks	2.57	1.45–4.55	0.001			
Social distancing	1.78	1.09–2.91	0.02			
Reported practice of COVID-19 prevention measures						
Wearing face masks	1.92	1.20–3.07	0.006			
Handwashing	2.29	1.31–4.00	0.004			
Social distancing	0.93	0.61–1.41	0.7			
Concerned about community spread	1.05	0.70–1.56	0.8			
Concerned about getting infected	1.00	0.64–1.53	1.0			
Confident that vaccines are safe	23.63	12.47–44.76	<0.001	28.14	13.89–57.01	<0.001
Drivers of decision to get vaccinated						
Recommendation from Ministry of Health	2.97	1.96–4.49	<0.001			
Recommendation from doctor	1.08	0.73–1.61	0.7			
Vaccine available free of charge	3.63	2.26–5.85	<0.001	3.83	2.05–7.15	<0.001
Ease of getting the vaccine	2.94	1.82–4.74	<0.001			
Recommendation from World Health Organization	2.88	1.56–5.31	0.001	3.12	1.41–6.92	0.005
Country where vaccine is produced	0.38	0.23–0.63	<0.001	0.47	0.24–0.95	0.04

OR: Odds ratio. aOR: Adjusted odds ratio. CI: Confidence interval. Ref: Reference.

## Data Availability

The data presented in this study are available on request from the corresponding author. The data are not publicly available to ensure confidentiality of responses.
